# Ontogeny of Skin Stem Cells and Molecular Underpinnings

**DOI:** 10.3390/cimb46080481

**Published:** 2024-07-28

**Authors:** Iasonas Dermitzakis, Despoina Dimitria Kampitsi, Maria Eleni Manthou, Paschalis Evangelidis, Efstratios Vakirlis, Soultana Meditskou, Paschalis Theotokis

**Affiliations:** 1 Department of Histology-Embryology, School of Medicine, Aristotle University of Thessaloniki, 54124 Thessaloniki, Greece; iasonasd@auth.gr (I.D.); dkampitsi@auth.gr (D.D.K.); mmanthou@auth.gr (M.E.M.); sefthym@auth.gr (S.M.); 2 Hematology Unit-Hemophilia Centre, 2nd Propedeutic Department of Internal Medicine, Hippocration Hospital, Aristotle University of Thessaloniki, 54642 Thessaloniki, Greece; pascevan@auth.gr; 3 First Department of Dermatology and Venereology, School of Medicine, Aristotle University of Thessaloniki, 54643 Thessaloniki, Greece; svakirlis@auth.gr

**Keywords:** skin, stem cells, epidermis, hair follicle, sebaceous glands, sweat glands, molecular cues, signaling, ontogeny, homeostasis

## Abstract

Skin stem cells (SCs) play a pivotal role in supporting tissue homeostasis. Several types of SCs are responsible for maintaining and regenerating skin tissue. These include bulge SCs and others residing in the interfollicular epidermis, infundibulum, isthmus, sebaceous glands, and sweat glands. The emergence of skin SCs commences during embryogenesis, where multipotent SCs arise from various precursor populations. These early events set the foundation for the diverse pool of SCs that will reside in the adult skin, ready to respond to tissue repair and regeneration demands. A network of molecular cues regulates skin SC behavior, balancing quiescence, self-renewal, and differentiation. The disruption of this delicate equilibrium can lead to SC exhaustion, impaired wound healing, and pathological conditions such as skin cancer. The present review explores the intricate mechanisms governing the development, activation, and differentiation of skin SCs, shedding light on the molecular signaling pathways that drive their fate decisions and skin homeostasis. Unraveling the complexities of these molecular drivers not only enhances our fundamental knowledge of skin biology but also holds promise for developing novel strategies to modulate skin SC fate for regenerative medicine applications, ultimately benefiting patients with skin disorders and injuries.

## 1. Introduction

The skin, also known as the integumentary system, is the largest organ of the human body, serving as a protective barrier between the internal body and the external environment [1]. It consists of three main layers: the epidermis, dermis, and subcutaneous tissue [2]. The outermost layer of the skin, the epidermis, provides waterproof protection and houses the melanocytes responsible for skin pigmentation. It also contains keratinocytes, which produce keratin for strength and elasticity [3,4,5,6]. Beneath the epidermis lies the dermis, which is composed of connective tissues, blood vessels, nerves, and appendages like hair follicles and sweat glands. The dermis provides structural support and regulates temperature through blood flow and sweat production [7,8]. The deepest layer of the skin, the subcutaneous tissue, consists of adipocytes that insulate the body and serve as an energy reserve. It also connects the skin to underlying muscles and bones [9].

The skin plays a crucial role in maintaining homeostasis within the human body. It accomplishes this by upholding a stable internal environment despite external influences [10]. Interestingly, the skin possesses its own homeostatic mechanism. Homeostasis, in this context, refers to its ability to regulate the skin barrier’s functionality by adjusting the balance between shedding and generating new skin cells [11]. In this context, skin layers assume a central role by harboring SCs that progressively differentiate into distinct cell types based on requirements [12]. In particular, bulge SCs and those in the interfollicular epidermis, infundibulum, isthmus, and sebaceous glands comprise the primary population of skin SCs [13,14,15,16]. Despite tissue SCs emerging during embryogenesis, the specific origin of the majority of adult SCs remains uncertain. The mammalian skin represents one of the most studied epithelial systems housings SCs. Likewise, the origins of most SC populations present in the adult epidermis remain unknown [17].

In numerous tissues, the development and maintenance of cells are meticulously governed by specific molecular signals [18,19,20]. The functionality of skin SCs is also influenced by molecular cues [21]. Specifically, their intricate and pivotal regulation by molecular drivers plays a crucial role in determining tissue homeostasis, repair, and regeneration. Effective modulation is essential in preserving the delicate balance between the quiescence, self-renewal, and differentiation of skin SCs, ensuring skin integrity and functionality [22]. The ontogeny of skin SCs, referring to their developmental origin and the regulatory mechanisms governing their behavior, is a topic of great interest and significance in regenerative medicine and developmental biology [23,24]. Interestingly, there is a growing interest in SCs due to their potential role in skin maintenance and disease. Any disturbances in these molecular cues have the potential to result in a range of skin disorders and cancer [25,26]. A comprehensive understanding of their function could provide valuable insights for preserving skin health [27,28].

The current review aims to explore the ontogeny of skin SCs, elucidating the intricate network of molecular signals guiding their fate and underscoring their pivotal contribution to skin homeostasis. Through a comprehensive synthesis of the available literature, we meticulously examine the molecular cues responsible for preserving SC stemness and regulating the delicate equilibrium between SC quiescence and activation. Ongoing advancements in this area of study not only augment our comprehension of skin biology but also present significant opportunities for pioneering therapeutic approaches to leveraging the regenerative capacity of skin SCs towards managing diverse skin pathologies.

## 2. Investigating Interfollicular Epidermal SCs: Key Players in Skin Homeostasis

### 2.1. Interfollicular Epidermal SC Proliferation and Differentiation

The interfollicular epidermis (IFE) is a multilayered squamous epithelium serving as the outermost layer of the skin. It primarily comprises four strata: the basal, spinous, granular, and cornified layers. The homeostasis of the IFE is intricately defined by the equilibrium between keratinocyte shedding from the skin surface through exfoliation and the self-renewal of IFE stem cells (IFESCs) accompanied by their differentiation [29,30]. The differentiation of IFESCs is considered a gradual process, as indicated by the stratification of the epidermal layers. Throughout differentiation, basal cells detach from the basement membrane, cease proliferation, and progress towards the final differentiation stage as they migrate outward across the layers of the epidermis. In human skin, IFESCs are situated in the basal layer at the apex of epidermal papillae in regions such as the breasts, foreskin, scalp, palms, and soles [31,32]. The lifespan of keratinocytes in humans is approximately 40–56 days [33]. Three hypotheses have been proposed regarding the heterogeneity among basal IFE cells and their proliferative and differentiative capacities: the hierarchical model, the stochastic model (also known as the single progenitor model), and the concept of two distinct SC populations [34,35,36].

The hierarchical model emerged from the findings of retroviral clonal marking in mouse skin, proposing a structure where keratinocytes are organized into columnar formations with hexagonal surfaces [34]. These columns traverse all the epidermal layers, forming autonomous epidermal proliferative units (EPUs) [37]. At the base of each EPU lies a solitary slow-cycling IFESC along with multiple secondary proliferative or transit-amplifying cells (TACs). The IFESCs undergo asymmetric division to replenish themselves and generate TACs, which subsequently commit to differentiation after several cell divisions, generating various suprabasal cell types within the corresponding EPU. This hypothesis supports the observed heterogeneity among basal layer cells in their proliferation and undifferentiated characteristics, evidenced by differential integrin expression levels within the basal layer [38,39]. However, subsequent investigations utilizing mutagens in mice have challenged the hierarchical model, revealing the non-hexagonal surface configurations of EPUs and the migratory capability of SCs between different EPUs [40]. Moreover, quantitative clonal studies in mice have shown a progressive reduction in the number of EPUs over time during homeostasis despite an increase in the size (cell count per EPU), casting doubt on the hierarchical EPU model [35].

The second theory encompasses the stochastic model, predicated on a singular population of SCs that alternate between division and differentiation with an equal potential [35]. This model does not apply the TAC state and is believed to be more adaptable to environmental fluctuations. It is yet to be determined that the cells upon which the stochastic model is based do not comprise another subpopulation within the heterogeneous basal SC population described by the hierarchical model [22]. Recently, a study employing cell-cycle distribution analysis, lineage tracing, and live imaging in mice confirmed the presence of a singular proliferative population, contradicting the hierarchical model except for the interscale region in tail skin, where the hierarchical model holds [41]. Another research endeavor utilizing the quantitative modeling of clonal fate data in mice proposed the 2xSC model, conclusively establishing the existence of two distinct SC populations: the slow-cycling SCs (also referred to as label-retaining SCs, LRSCs) and the fast-cycling SCs (also referred to as non-label-retaining SCs, non-LRSCs) [36].

The non-LRSCs were traditionally believed to be responsible for maintaining homeostasis, while LRSCs were thought to be activated during wound repair. This pattern elucidates the reported heterogeneity among SCs and mirrors what has been observed in other tissues such as the cornea, hematopoietic system, central nervous system, hair follicles, and muscle [42,43,44,45,46,47]. Subsequent investigations have validated the presence of two distinct SC populations, revealing that the LR and non-LR SCs contribute to both homeostasis and wound repair within different skin compartments characterized by varying regeneration frequencies [35]. This distinction was established by utilizing the H2B-GFP pulse-chase system in the skin of adult mice. The regions containing non-LRSC exhibit high blood vessel organization, notably associated with pericytes that promote SC self-renewal. This observation corresponds with the significant proliferation capacity of non-LRSCs [48,49]. Furthermore, non-LRSCs express molecules involved in blood vessel formation, underscoring an active cross-talk between the epidermis and blood vessels. Notably, previous studies have identified numerous IFESC populations displaying proliferation rates comparable to those of LRSCs, leading researchers to conclude that the LRSC population is distinct.

SCs can undergo either symmetric cell divisions (SCDs) or asymmetric cell divisions (ACDs). By inducing genetic labeling in mice, it has been estimated that approximately 84% of all the divisions were ACDs, while another 8% resulted in SCDs [35]. ACDs allow SCs to both self-renew and differentiate into keratinocytes, whereas SCDs result in a notable increase in the SC population. During ACDs in the embryonic development of the epidermis, the mitotic spindle orientation is believed to be perpendicular to the basement membrane, giving rise to an undifferentiated basal cell that remains attached to the basement membrane and a second committed daughter cell that detaches from it [50]. However, subsequent studies utilizing in vivo live-imaging microscopy to track the dynamics of SCs over time revealed that in adult skin, all the divisions were horizontally oriented towards the basement membrane [51]. Whether vertically oriented divisions play a role in epidermal homeostasis remains unresolved and subject to ongoing discussion.

### 2.2. Molecular Cues Regulating IFESCs

Keratinocyte attachment to the basement layer is facilitated by hemidesmosomes and focal adhesions composed of integrins. The basement membrane plays a crucial role in regulating IFESC function due to the specialized microenvironment it provides. β-integrin serves as a marker for the isolation of IFESCs [31,32,52,53]. In vitro testing suggests that β1-integrin plays a pivotal role in SC proliferation, as high expression levels are associated with keratinocytes capable of forming colonies and identified as LRSCs in the basal layer of the epidermis. Considerable discrepancies exist between the findings from in vitro and in vivo studies regarding the involvement of β-integrin in maintaining IFESCs. Studies involving mice with β1-integrin deletions reported impaired cellular adhesion to the basement membrane, the disturbed proliferation of IFESCs, as well as IFE disorganization and hyperplasia [54,55]. However, despite this phenotype, knockout mice were reported to survive for up to one year, proving that IFESCs could still maintain skin homeostasis; thus, the absence of β1 integrin does not necessarily result in SC exhaustion [54].

In the same line, cell–extracellular matrix attachment is crucial in establishing the SC niche. *COL17* exhibits high expression levels in the apical rete ridge regions, where SCs reside [56]. Functioning as a transmembrane protein, COL17 contributes to the formation of hemidesmosomes and mediates the interactions of SCs with their microenvironment to regulate epidermal homeostasis [57]. The depletion of *COL17* has been associated with a decline in adult human IFESCs in a Wnt signaling-independent way [56,57,58]. Regarding the cytoskeleton, the actin cytoskeleton is intricately involved in promoting differentiation influencing the activity of the SRF transcription factor [59]. SRF targets genes such as *FOS* and *JUNB*. SC differentiation was suppressed upon the knockdown of the SRF/MAL complex in human IESCs. On the other hand, the upregulation of *MAL* activated *SRF* activity and triggered the synthesis of involucrin, a marker of differentiation. Actin filament remodeling is facilitated by RAC1, a member of the Rho family of GTPases regulated by the tyrosine receptor kinase cascade, including the Egfr pathway [60]. The loss of *RAC1* in human IFESC culture leads to the organization of actin filaments into a pattern characteristic of TACs, resulting in diminished proliferative properties. Knockout mice fail to maintain IFE homeostasis due to the depletion of SCs. Consequently, RAC1 is indispensable for preserving the proliferative capacity of IFESCs.

The primary cilium is another cytoskeleton-related structure that impacts the function of IFESCs in addition to integrins and actin filaments mentioned previously. Primary cilia are nonmotile organelles constructed with microtubules that receive signals from the extracellular matrix [61]. The function of primary cilia is associated with various signaling cascades. Lineage-tracing in mice studies has demonstrated that mutations in *Ift88* and *Kif3a* genes involved in cilia formation result in irregular proliferation and relocation of hair follicle bulge SCs to the IFE [62]. When exposed to DELTA signals from adjacent cells, human keratinocytes in the IFE are induced to undergo differentiation unless they exhibit high levels of *DELTA1* expression [63]. IFESCs display elevated *DELTA1* expression compared to surrounding cells. Increased *DELTA1* expression in SCs may safeguard their proliferative capacity by inhibiting Notch signaling. This discovery indicates the impact of Notch signaling on the fate of IFESCs. While the functions of this pathway have been extensively studied in embryonic development, there is a growing body of evidence suggesting that Notch signaling continues to govern cell fate in adult tissues that undergo self-renewal or regeneration.

**Figure 1:**
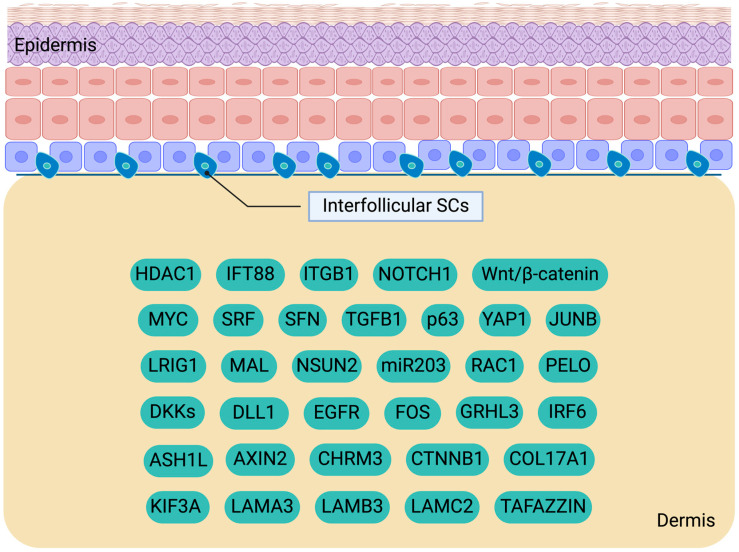
Molecular cues orchestrating the maintenance, proliferation, and differentiation of interfollicular epidermal stem cells (IFESCs). IFESCs are located in the basal layer of the epidermis. Created with BioRender.com.

It is noteworthy that, aside from the signals originating from the basement membrane and the extracellular matrix, IFESCs autonomously dictate their fate by secreting factors that either support self-renewal or stimulate differentiation. *AXIN2*, a target gene of the Wnt/β-catenin pathway, has been identified as a marker for tracking IFESCs through mouse lineage tracing and quantitative clonal analyses [64]. Through the utilization of dual-labeling RNA in situ hybridization in mice, IFESCs expressing *Axin2* were found to release Wnt signals, such as WNT4 and WNT10, which facilitate self-renewal, as well as Wnt inhibitors (namely DKKs). These data suggest an autocrine mechanism by which IFESCs establish a self-niche. Furthermore, it indicates that as SCs undergo differentiation and move away from the basal layer microenvironment, they experience reduced exposure to the Wnt signals secreted by basal layer cells and heightened exposure to the Wnt inhibitors unleashed by suprabasal cells, which promote differentiation (Figure 1).

Experiments involving human keratinocytes have revealed elevated cytoplasmic β-catenin levels in IFESCs compared to reduced levels in TACs [65]. There was no apparent correlation between β-catenin expression and cell–cell adhesion. Additionally, IFESCs exhibited more intense *TCF/LEF* activity than TACs. These findings verify the link between the Wnt/β-catenin pathway and the maintenance of stemness. The retroviral transduction of stabilized β-catenin significantly augments the population of proliferative SCs without affecting TACs. Conversely, the induction of a dominant-negative form of β-catenin leads to a suppression of cell proliferation. These results indicate the critical role of the Wnt/β-catenin pathway in preserving the IFESC pool. Furthermore, transcription factor GRHL3 has been implicated in inhibiting IFESC expansion by suppressing the Wnt pathway in these cells [66]. These findings underscore the significance of the Wnt/β-catenin pathway in regulating IFESC function.

Interestingly, initial experiments regarding mutations resulting in the loss-of-function of β-catenin, *Lef1*, or *Porcn* have been observed to impact hair follicles without causing abnormal phenotypes in the IFE [67,68,69]. Numerous hypotheses have been proposed to elucidate the absence of an abnormal IFE phenotype in these studies [70]. A more comprehensive examination of the loss-of-function experiments revealed that the Wnt/β-catenin pathway might not have been completely inactivated, suggesting that the residual signaling activity levels could have been adequate to meet the epidermal requirements and maintain homeostasis. Furthermore, unidentified molecules within the IFE might have overlapping functions with Wnt/β-catenin signaling.

An intriguing hypothesis further underscores the significance of the Wnt/β-catenin pathway in the IFESC function [71,72]. These observations prompt the assumption that the Wnt/β-catenin pathway exerts its influence throughout the epidermis, and the level of its signaling activity, in conjunction with other signals, determines the fate of SCs. According to this hypothesis, weak Wnt/β-catenin activity specifies the differentiation into keratinocytes, median activity leads to sebocyte differentiation, and increased activity promotes hair follicle cell differentiation. Hence, it is postulated that epidermal SCs can generate all the epidermal lineages under specific conditions [73]. This theory is substantiated by the observation that following injury, a Wnt-associated signal facilitates the formation of new hair follicles using SCs outside the hair follicle SC niche [74]. Conversely, it has been demonstrated that the absence of Wnt/β-catenin in hair follicular bulge SCs impedes their differentiation into hair follicular cells, redirecting them towards differentiation into epidermal cells [67].

While Wnt/β-catenin maintains the stemness of IFESCs, c-MYC facilitates their transition into the transit-amplifying state [75]. Reports indicate that *c-Myc* expression can be induced by β-catenin in mice, suggesting a potential feedback loop that regulates the balance between IFESCs and TACs [65,76]. c-*MYC* is classified as a proto-oncogene, and extensive studies have highlighted its role in promoting cell proliferation. Specifically, c-*MYC* expression in humans is found to be upregulated by EGF, leading to enhanced proliferative effects [77]. The induction of LRIG1, an inhibitor of the EGFR, restricts its expression and consequently limits keratinocyte proliferation. Moreover, the activation of *Myc* in mice induces the activation of *Misu*, a gene for an RNA methyltransferase that promotes DNA synthesis [78]. Contrary to previous beliefs, MYC has also been reported to drive the differentiation of SCs [75]. Subsequent investigations have confirmed the positive role of MYC in differentiation, proposing two potential mechanisms [79]. The first mechanism suggests that MYC regulates diverse genes across different cell types, with its effects depending on the cellular context. The second mechanism indicates that MYC activity is complex and not a simple on/off switch; its effects are influenced by the activation’s degree, duration, and timing. Temporarily elevated MYC levels are thought to enhance proliferation, whereas sustained high levels of MYC drive SCs towards the TAC state. MYC functions by downregulating genes associated with cell–extracellular matrix adhesion, promoting cell migration and differentiation [80,81]. Additionally, MYC facilitates euchromatin formation, enabling the activation of dormant SCs [79].

Thus, the properties of proliferation are not solely regulated by genetic factors but also by epigenetic mechanisms involving histone modifiers. Among these, ASH1L, a SET-domain histone lysine methyltransferase belonging to the trithorax group of proteins, affects SC behavior [82]. Its deficiency results in an elevated proliferation rate of IFESCs, subsequently leading to hyperplastic epidermis and skin lesions, a fascinating aspect of SC biology that warrants further investigation. An interesting mechanism supported by experiments in human samples suggests that P63 acts by downregulating *HDAC1* [83]. The expression of P63 is abundant in IFESCs and serves as a specific marker for their identification, considering that TACs exhibit only low levels [83]. This observation implies that P63 plays a critical role in maintaining the stemness of IFESCs [84]. The loss of *p63* in mice results in a pronounced phenotype characterized by a monolayered epidermis due to premature proliferative restriction in both IFESCs and TACs [85,86]. Notably, miR-203 has been documented to promote differentiation in vitro by targeting and inactivating ΔNp63 [85]. Lastly, *14-3-3σ* induces *p63* expression, as evidenced by the heightened levels of P63 in IFESCs and the accelerated proliferation rate of IFESCs in *Sfn^+/Er^* mice [87].

Another pathway involved in the regulation of SC properties is Hippo signaling. The Hippo pathway comprises a kinase cascade that leads to the phosphorylation and inactivation of the YAP and TAZ transcription factors [88]. In a quiescent Hippo state, YAP and TAZ remain unphosphorylated, enabling their translocation into the nucleus to drive the expression of target genes responsible for cell proliferation, survival, and migration. The subcellular localization of YAP and TAZ shifts according to the differentiation stage of cells; they exhibit nuclear localization in IFESCs, while they display cytoplasmic localization in suprabasal keratinocytes. This translocation is associated with SC detachment from the basement membrane during differentiation in a β1-integrin-associated way. YAP’s function is facilitated by laminin 332 preserving IFESCs [89]. This signaling pathway is crucial for homeostasis, and its dysregulation can lead to restricted SC proliferation, exhaustion, skin damage, and delayed repair following injuries. In addition to its interaction with P63, the disruption of 14-3-3σ also leads to increased nuclear levels of YAP1, prompting SC proliferation over differentiation [87]. This effect was demonstrated in experiments with Sfn^+/Er^ mice, which exhibited epidermal hyperplasia. The heightened proliferation frequency observed in IFESCs of mice homozygous for mutations in the *14-3-3σ* gene is also evident in mice carrying a missense mutation in the *Irf6* gene [90]. This increased proliferation is attributed to the failure of IFESCs to transition from proliferation to differentiation. These findings highlight the novel and significant roles of *IRF6* and *14-3-3σ* in regulating the proliferation–differentiation switch in SCs.

Except for the aforementioned, ribosome-related mRNA surveillance, which ensures precision in protein synthesis, could regulate the function of IFESCs. PELOTA (also known as PELO) is an evolutionarily conserved factor essential for the mRNA quality control mechanism [91]. This process is crucial for epidermal homeostasis. In mice, the conditional deletion of *Pelo* in LRIG1^+^ IFESCs resulted in overproliferation and aberrant differentiation. Another significant molecule is LRIG1, which functions as an IFESC marker crucial for maintaining their quiescence by inhibiting the Egfr pathway [92]. The knockdown of *LRIG1* results in increased proliferation frequency and clonal enlargement in cultured human keratinocytes [77]. Furthermore, the population of LRSCs is reduced in *Lrig1*-null IFE of adult mice [93]. Maintaining the properties of SCs is essential to ensure homeostasis and prevent depletion. The Tgf-β signaling pathway is crucial in maintaining the nature of SCs [94]. The Tgf-β cascade stimulates the expression of K#5 and K#14, which serve as the markers of IFESCs. Recent research indicates the involvement of acetylcholine in regulating epidermal differentiation, suggesting the potential therapeutic benefits of cholinergic medications in dermatological conditions [95]. Remarkably, the loss of the M3 muscarinic acetylcholine receptor in *Chrm3^−/−^* mice led to the expansion of SCs and a decrease in differentiated cells, indicating that *Chrm3* guides IFESCs towards differentiation. The molecular signals and corresponding genes that regulate the properties of IFESCs are summarized in Figure 1 and Table 1.

## 3. Insights into Hair Follicle SCs: Bulge and Hair Germ Dynamics

### 3.1. Identification and Characteristics of Hair Follicle SCs

The hair follicle (HF) contributes to the formation of the pilosebaceous unit in conjunction with the sebaceous gland and the arrector pili muscle [97]. Anatomically, HF is segmented into three distinct compartments: the infundibulum, situated between the IFE and the opening of the sebaceous duct; the isthmus extending from the sebaceous gland opening to the bulge; and the bulge representing the deepest static segment of the HF. At the base of the HF resides a specialized cluster of mesenchymal cells referred to as the dermal papilla (DP) [98]. Over a lifetime, HF undergoes cyclical phases of degeneration and regeneration, transitioning from the growth phase (anagen) to the transitional phase (catagen) and finally to the resting phase (telogen) [99]. Prior to commencing a new cycle, the HF must regenerate its hair bulb. Consequently, the lower portion of the HF experiences continual renewal, while the upper portion remains relatively stable. This regeneration of the hair bulb is orchestrated by hair follicle stem cells (HFSCs). In terms of their functionality, HFSCs exhibit distinct behaviors during the different hair growth phases. Anagen is typified by robust proliferation and growth, catagen is associated with degeneration through apoptosis, while quiescent HFSCs characterized telogen.

Initially, HFSCs were specifically denoted as the cells residing in the mouse bulge, known as bulge stem cells (BuSCs) [44,100]. These cells were meticulously identified through nucleotide analog pulse-chase experiments. They were distinguished as slowly proliferating LR cells and displayed the capacity to regenerate both the IFE and the HF, as evidenced by grafting experiments [16,44]. Their tracking involved the use of markers CD34 and integrin-α6. Through microdissection and flow cytometry utilizing CD34 or K15-GFP markers, the isolation of BuSCs and their subsequent in vitro culture was made feasible [16,101,102,103,104]. Consequently, it was unveiled that BuSCs possess the ability to generate expansive colonies comprising cells that retain their proliferative characteristics. The transplantation of a colony originating from a single BuSC resulted in the development of all HF lineages [101,105]. Nonetheless, lineage-tracing techniques employing Tet-o-H2B-GFP or markers such as K15, LGR5, or K19 have demonstrated that the maintenance of homeostasis is not solely attributed to BuSCs but also involves cells situated in the hair germ, identified as hair germ stem cells (HGSCs) [16,106,107,108].

During homeostasis, HFSCs exclusively contribute to the renewal of HF lineages [16,106,107]. However, following traumas, they also give rise to IFE cells [109,110]. This discovery suggests that distinct SC populations in adults are responsible for regenerating the HF and IFE, a demarcation that becomes apparent during hair placode development. This evidence was gleaned from embryonic lineage tracing studies, which revealed that HFSCs originated from embryonic SHH^+^ and SOX9^+^ cells [111,112]. At the same time, IFESCs do not seem to derive from cells displaying these markers. Although the structure of the HF bulge and the expression of HFSC markers are not evident until the third week of postnatal life in mice, H2B-GFP pulse-chase studies conducted at E18.5 demonstrate that the slow-cycling potential HFSCs emerge during embryogenesis and transition to adult HFSCs by P2 [101,112]. Subsequently, these cells are exclusively located in the bulge and the HG upon the completion of HF development.

Quantitative H2B-GFP label-retention experiments have revealed that during homeostasis, approximately 94% of HFSCs complete at least one cell division per hair cycle, with a majority undergoing three or more proliferations [113]. Lineage-tracing of individual BuSCs has elucidated their capacity to migrate from the bulge to the HG region [114]. Despite over 90% of LGR5^+^ cells being co-labeled with CD34 and exhibiting quiescent characteristics similar to other BuSCs, studies have shown that LGR5^+^ cells residing within the HG region display a higher proliferative activity [107]. Generally, both BuSCs and HGSCs comprise subsets of cells that exhibit low division rates in the preceding hair cycle, as indicated by H2B-GFP label-retention experiments [114,115]. Further elucidation of the distinct roles of both BuSCs and HGSCs during homeostasis necessitates the generation of new inducible CRE mice, as existing models are not specialized for a distinct region [22].

### 3.2. Molecular Mechanisms Governing Activation and Quiescence of HFSCs

The utilization of bromodeoxyuridine pulse demonstrated that the first HFSCs to become mobilized were the HGSCs, followed by the activation of BuSCs [99]. This observation indicates a two-step process for activation. Towards the end of telogen and the commencement of anagen, signaling cues originating from the DP stimulate the activation of HGSCs [99,103,108,116]. Lineage-tracing investigations have unveiled that HGSCs give rise to the hair matrix, comprising TACs [99,117,118,119]. TACs represent an intermediary cell population between SCs and terminally differentiated cells, exhibiting a unique expression profile distinct from both BuSCs and HGSCs. TACs exhibit rapid proliferation, undergo differentiation after several rounds of division, and contribute to the regeneration of the hair shaft and inner root sheath (IRS). Conversely, BuSCs migrate downward, occupying the zone between the bulge and the matrix, thereby forming the outer root sheath (ORS) [115,119]. This process necessitates SOX9, as the absence of this transcription factor impedes the formation of the ORS. In the absence of SOX9, the establishment of the adult HFSC population is unsuccessful.

Lineage-tracing techniques employing H2B-GFP, conventional lineage-tracing methods, and nucleotide pulse-chase assays have enabled researchers to investigate the fate of HFSCs [115]. During the anagen phase, cells in the ORS’s upper region maintain their slow-cycling characteristics, while those in the lower ORS exhibit high proliferative activity. Notably, a small number of ORS cells evade apoptosis during the catagen phase. These surviving ORS cells give rise to the degenerated bulge and HG cells, preparing for the subsequent hair cycle. ORS cells nearest to the bulge, having undergone fewer cell divisions, are deemed to be recycled to sustain the long-term maintenance of the pool, while cells positioned further away give rise to the HGSCs [99]. Highly proliferative cells adjacent to the matrix that have surpassed a certain threshold retain the ability to return to the bulge but lose their proliferative properties [115]. Despite maintaining typical BuSC characteristics, they do not function as efficient BuSCs. Intriguingly, these cells contribute to niche formation by providing physical support for the hair structure. Additionally, they secrete BMP6 and FGF18, which are known to regulate SC quiescence [101]. This intricate mechanism underscores the essential role of the bulge niche in maintaining HFSC quiescence, yet not the conservation of their stemness [115].

**Figure 2:**
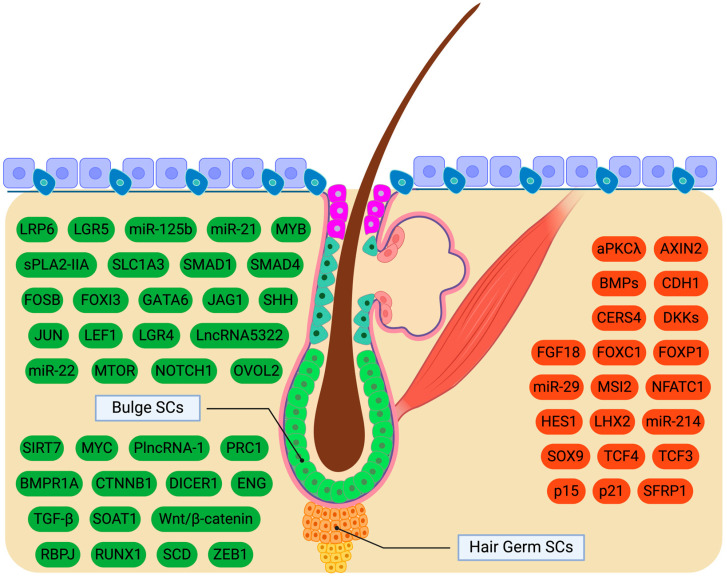
Molecular regulators governing the transition between quiescence and the activation of hair follicle stem cells (HFSCs). Specifically, green-colored signals activate HFSCs, whereas red-colored cues preserve HFSC quiescence. HFSCs include bulge SCs (green cells) as well as hair germ SCs (orange cells). Created with BioRender.com.

Given that transcriptional alterations leading to the activation of BuSCs resemble those observed during HGSC activation, the precise mechanism driving the delayed activation of BuSCs remains incompletely understood. It has been suggested that the heightened sensitivity of HGSCs may be attributed to intrinsic factors [101,120]. Considering the expression of BMPs by BuSCs, it is also plausible to speculate that BMP inhibitors play a role in this process. Notably, BMP antagonists are upregulated in the dermis during the telogen phase, promoting the transition of the hair cycle to anagen [121,122]. It is conceivable that the elevated expression of dermal BMP inhibitors may have a more pronounced effect on HGSCs due to their proximity to the DP compared to BuSCs [99]. According to this model, the reason behind the slow proliferation of BuSCs during anagen, a phase where the dermal papilla is absent from the niche, remains unclear. The BMP pathway maintains HFSCs in a quiescent state by suppressing the Wnt/β-catenin pathway [120,123]. Thus, BMP signaling represses the activation and proliferation of HFSCs, keeping them in a dormant state, while the inhibition of BMP leads to the premature activation of HFSCs (Figure 2).

In *aPKCλ* conditional knockout mice, the quiescence of HFSC was also compromised, as evidenced by the reduced expression levels of the factors known to induce quiescence, such as FGF18 and BMP6 [124]. Similarly, FOXP1 maintains the quiescence of HFSCs by regulating FGF18 levels. This was illustrated in *Foxp1* deficient mice, where HFSCs experienced premature activation [125]. Another transcription factor, namely FOXC1, facilitates cross-talk among diverse signaling cascades. FOXC1 directly regulates target genes and indirectly influences the microenvironment. Specifically, FOXC1 promotes the expression of the genes involved in maintaining quiescence, including *NFATC1* and *BMP2* [126]. Moreover, by upregulating E-cadherin expression, FOXC1 maintains the population of older bulge cells, which in turn produce BMP5 and FGF18 to sustain the quiescent state of the bulge [127].

In addition to the factors governing the quiescence of HFSCs, the regulators ensuring the optimal activation and proliferation of HFSCs are also paramount. HFSCs express a unique combination of transcription factors responsible for their specialized functions. Firstly, HES1 serves as a transcription factor that suppresses the Notch pathway [128]. The Notch/RBP-J cascade plays a crucial role in inhibiting the differentiation of HFSCs into IFE cells while promoting their differentiation into HF lineage cells [129]. This function allows HES1 to maintain stemness. HES1 exhibits high and consistent levels in the lower bulge and the HG as the transition from telogen to anagen progresses [128]. Reduction in HES1 levels disrupts differentiation and cell proliferation within the HF, ultimately limiting the characteristics of the SCs. Mice with a *Hes1* knockout demonstrate diminished HF renewal, indicative of HFSC depletion. Comparative transcriptome analyses have revealed HES1’s involvement in activating the Shh pathway. These data indicate a potential interplay between the Notch and Shh pathways, mediated by HES1. Within mice HF, the expression of *Hes1* was proposed to be linked to alterations in the transcription of the gene for the JAGGED-1 ligand. Specifically, decreased HES1 levels restrict ligand–receptor interactions, downregulating the HES1 pathway.

Wnt/β-catenin signaling is regulated by a subset of transcription factors that can either enhance or inhibit its effects. For instance, the TCF/LEF transcription factor family consists of members that may act as the antagonists or activators of the Wnt signaling target genes [130]. In postnatal life, TCF3/4 inhibits the Wnt pathway target genes, suppressing HFSC activation and differentiation [131]. Their impact is mediated through β-catenin binding. In contrast, LEF1 functions by activating the Wnt signaling pathway [132]. Notably, *Lef1* is prominently expressed during the anagen phase but diminishes during other hair cycle phases in vivo. Upon stimulating rat HFSC differentiation in vitro through the co-culture of mice HFSCs with DP cells, LEF levels significantly increased and induced the expression of *c-Myc* and *Jagged1*. Concurrently, NOTCH1 and β-catenin levels were elevated, confirming the activation of the Notch and Wnt cascades. It is noteworthy that a well-coordinated interplay of various Wnt and Bmp factors binding to super-enhancers dictates HFSC functions [133]. Specifically, the replacement of TCF3/4 by LEF1 stimulated HFSC differentiation into TACs, while the co-binding of RBPJ and pSMAD1 with LEF1 led to the differentiation into IRS or hair shaft cells, respectively. HF regeneration exhibited impairment in mice with conditional knockout of *Foxi3*, underscoring the pivotal role of FOXI3 in SC activation [134]. FOXI3 plays a crucial role in activating the Wnt pathway and facilitating the expansion of TACs. The precise mechanism by which FOXI3 enhances Wnt signaling remains uncertain.

Another role of the Wnt/β-catenin pathway in regulating HFSC function has also been proposed. Serine-threonine kinase suppressors stimulate CK1 to phosphorylate AXIN2 [135]. Phosphorylated AXIN2 binds to β-catenin, leading to its ubiquitination and subsequent degradation. *AXIN2*, identified as a Wnt-target gene, is a marker for quiescent BuSCs. Using *Axin2-lacZ* mice, it was discovered that outer bulge cells produce Wnt signals autonomously, thereby preserving HFSC potency. However, the secretion of Wnt inhibitors like DKKs and SFRP1 maintains inner BuSCs in a quiescent state during the telogen phase. These findings emphasize the importance of Wnt/β-catenin signaling regulation in maintaining BuSCs, as the lack of such a control mechanism results in the premature activation of BuSCs and depletion of the bulge. *LGR5* represents another Wnt pathway target gene that is intricately involved in the regulation of HFSCs [107,136]. It is significantly expressed in HFSCs, making it a valuable marker for targeting SCs. Specifically, LGR5 is an enhancer of the Wnt pathway and supports SC self-renewal [137]. Moreover, in BuSCs, LGR5 upregulates the Shh pathway to enhance proliferation and differentiation during growth [138]. Another member of the same family, namely LGR4, promotes the activation of HFSCs [139]. Its depletion suppresses both mTOR and Wnt pathway activities that stimulate Bmp signaling function, inhibiting HFSC activation and delaying the transition to the anagen phase.

Similarly, RUNX1 is essential for the activation and proliferation of HGSCs and BuSCs by inhibiting the cyclin-dependent kinase inhibitors P21 and P15 [140,141]. Insufficient levels of RUNX1 trigger the differentiation of HFSCs into sebocytes, resulting in the loss of their capacity for self-renewal [142]. Researchers propose an additional mechanism observed in other tissues, suggesting that RUNX1 influences the fatty acid composition of cell membranes by regulating the activity of *SCD1* and *SOAT1* [143]. SCD1 and SOAT1 are lipid enzymes that modulate the Wnt signaling pathway. The hypothesis that this mechanism is operative in HFSCs is supported by the presence of SCD1 and SOAT1 in these cells. Additionally, ENG serves as a receptor involved in the Tgf-β cascade [144]. TGF-β signaling can counteract the suppression of HFSC activation mediated by BMPs. *ENG* expression is modulated by certain heterodimers comprising β-catenin and SMAD4, pivotal players in the Wnt and Bmp pathways, respectively. A systemic decrease in *Eng* expression in mice resulted in the dysregulation of key hair cycle regulators. *Gata6* is also expressed in HGSCs during the transition from telogen to anagen in the adult murine skin [145]. Findings from in vivo and in vitro investigations in TACs suggest that GATA6 protects from the accumulation of DNA mutations, indicating its role in alleviating stress in rapidly proliferating cells.

The proto-oncogene *MYB* exhibits high expression levels in the lower bulge, the ORS, and the matrix [146]. Although MYB may not be essential for controlling the slowly dividing HFSCs within their niche, it is conceivable that c-MYB could be involved in activating quiescent SCs as they transit out of the niche. The identification of OVOL2 in the IFE, bulge, HG, and TACs within murine skin is noteworthy, underscoring the importance of HFSC migration for HF homeostasis [147]. OVOL2 is a transcription factor that impedes epithelial-to-mesenchymal transition (EMT) by enhancing *Zeb1* expression, as evidenced by live cell imaging and cell cycle analysis in mice. It was suggested that OVOL2 plays a role in migration and cell cycle progression, achieved through the suppression of EMT via *ZEB1* activation. Both in vitro and in vivo experiments demonstrated that insufficient OVOL2 in HFSCs exhibited compromised clonal production capabilities and structural impairment upon modifications in the microenvironment.

Besides transcription factors that modulate signaling cascades, other molecules also significantly contribute to HF homeostasis through deacetylation and post-transcriptional mRNA modification. These mechanisms will be further analyzed. Firstly, SIRT7 is a deacetylase associated with rRNA expression and cell aging [148]. It plays a critical role in ensuring timely entry into the anagen phase for BuSCs. The absence of SIRT7 results in the delayed activation of HFSCs, while its induction during telogen leads to a premature transition to anagen. In vitro studies have demonstrated that SIRT7 functions by facilitating the breakdown of NFATC1. NFATC1, a transcription factor, maintains SCs in a quiescent state. Furthermore, MSI2 is a protein with RNA-binding capabilities that is a component of the ribonucleoprotein complex, modulating mRNAs’ post-transcriptional editing [149]. Using gain- and loss-of-function mouse models, it has been observed that the loss of *Msi2* leads to the accelerated proliferation of HFSCs. The proposed mechanism by which MSI2 operates involves binding to *SHH* mRNA, thereby inhibiting its translation to sustain the quiescence of HFSCs. This hypothesis is supported by transcriptomic analysis and crosslinking immunoprecipitation-PCR experiments.

DNA regulatory elements, such as promoters, enhancers, and super-enhancers, play a crucial role in maintaining stemness by enabling or inhibiting access to transcription factors. The precise mechanism underlying their function remained largely unexplored until recently due to the lack of tools for monitoring enhancer effects [150]. Current studies have suggested super-enhancers’ involvement in governing HFSC stemness and quiescence. SOX9 facilitates the conversion of heterochromatin to euchromatin, allowing transcription factors such as LHX2 and NFATC to access the DNA. The function of LHX2 in preserving the growth and undifferentiated traits of HFSCs has been elucidated [151]. In particular, the lack of *Lhx2* rendered in mice HFSCs more prone to activation, leading to enhanced proliferation and differentiation. Likewise, the inhibitory role of NFATC1 in regulating the activation of BuSCs has been emphasized through gain- and loss-of-function studies [152]. The PRC1 represents an additional epigenetic modifier that plays a role in maintaining the proliferative properties of HFSCs [153]. Recent findings have shown that the absence of PRC1 in LGR5^+^ HFSCs reduces H2AK119Ub levels, potentially limiting HFSC proliferation. The loss of PRC1 in mice leads to upregulating genes associated with cell growth and suppressing HF lineage-specific genes, including *Shh*. The Shh pathway is crucial for both activating quiescent BuSCs and supporting their self-renewal during the growth phase of the hair cycle [117,154].

Non-coding RNAs, DICER, and long non-coding RNAs (lncRNAs) represent crucial intrinsic regulatory elements in HFSC function. To commence, miR-22 and miR-125b govern differentiation and sustain stemness in HFSCs [155,156,157]. The miR-29 exhibits predominant expression during telogen, and upon its suppression, the premature activation of HGSCs occurs, leading to an earlier onset of anagen (Figure 2) [158]. The effects of miR-29 may be mediated through blocking LRP6 and BMPR1A, which are recognized as miR targets. Besides, miR-214 suppresses the proliferation and differentiation of HFSCs by counteracting the Wnt pathway [159,160]. Moreover, in vitro experiments demonstrate that PlncRNA-1 enhances proliferation and differentiation in HFSCs by activating the TGF-β1 and Wnt signaling cascades while regulating miRNAs [161]. For instance, IncRNA5322 induces proliferation and differentiation by positively modulating miR-21, which promotes the phosphorylation of PI3K/AKT [162]. Furthermore, DICER is a component of the miRNA-induced silencing complex along with miRNAs [163]. *Dicer* deletion in BuSCs in mice results in impaired HF differentiation, potentially due to reduced keratin levels in the layers of HFs. TARBP2 is another component of the miRISC complex; however, mice lacking TARBP2 did not exhibit a similar phenotype to *Dicer* ablation.

In addressing the molecules involved in determining the fate of HFSCs, it is essential to consider the active role of metabolism in regulating HFSC functions. Metabolism is typically intertwined with the signaling cascades in HFSCs, including Bmp, Wnt, and Notch signaling pathways. Initially, the glutamine pathway is believed to facilitate HFSC activation, as the glutamate transporter SLC1A3 is crucial for timely HFSC activation and transition to the growth phase [164]. Lineage tracing studies in mice revealed that SLC1A3-positive cells within BuSCs contribute to long-term HF renewal [48]. Experiments involving *Slc1a3* ablation in mice suggest its involvement in mediating interactions between HFSCs, the IFE, and sebaceous gland SC niches [164]. Whether *Slc1a3* knockout primarily affects HFSCs or their niches remains uncertain. During anagen, TACs exhibit enlarged, highly active mitochondria with abundant cristae in contrast to the mitochondria of BuSCs [165]. The transcriptional profiling of enzyme expression indicates that TACs predominantly utilize aerobic respiration, while BuSCs engage in anaerobic respiration.

Notably, lactate production plays a fundamental role in HFSC activation, with its induction leading to rapid HFSC activation [166]. Lipid metabolism also appears to influence HFSC. The upregulation of phospholipase sPLA2-IIA in mice induces HGSC differentiation, mirroring the effects of mitogens and pro-proliferative transcription factors such as c-JUN and FOSB [167]. The prolonged overexpression of sPLA2-IIA ultimately leads to HFSC depletion. Additionally, Runx1, previously discussed, plays a role in lipid metabolism as well [143]. The perturbation of ceramide metabolism by downregulating *CerS4* in mice triggers the premature stimulation of HFSCs, resulting in a shift from the suppressive Bmp pathway to the activating Wnt pathway [168]. Notably, the knockout of the gene for ceramidase ACER1 does not result in any phenotype beyond the disorganization of K15 in the bulge region [169,170]. Investigating whether these findings are related to ceramide levels or the cascades regulated by the aforementioned enzymes is warranted. Molecular cues and genes involved in HFSC function are presented in Figure 2 and Table 2.

## 4. Potential of Isthmus, Infundibulum, and Sebaceous Gland SCs for Homeostatic Regulation

### 4.1. Distinctive SC Populations

The region of an HF over the bulge is divided into three segments: the isthmus, which is the narrow upward extension of the bulge; the sebaceous gland (SG), which is connected to the isthmus through the sebaceous duct; and the infundibulum forming the junction between the isthmus and the IFE [171]. The cellular population of the isthmus and the infundibulum remains relatively stable. However, to maintain homeostasis, SGs need to undergo continual regeneration. Sebaceous gland stem cells (SGSCs) are located in the outermost layer of the lobule and are anchored to the basal lamina, distinguishing the SG from the adjacent dermis [172]. SGSCs that detach from the basal region undergo differentiation and migration towards the central region of the gland, passing through the intermediate maturation zone before reaching the degeneration zone. Within the maturation zone, SG cells enlarge and gradually accumulate lipids in their cytoplasm [173]. In contrast, the degeneration zone consists of SG cells densely packed with lipid droplets, exhibiting karyopyknosis before rupturing and releasing lipid contents onto the epidermal surface through the sebaceous duct and the HF lumen.

Stem and progenitor populations have been identified in the upper region of the pilosebaceous unit. Initially, the junctional zone between the IFE and the upper isthmus is populated by LRIG1^+^ cells, constituting a multipotent SC population [93,174]. This population gives rise to cells of the IFE and the upper pilosebaceous unit, including the infundibulum and SG, but not HF. Further down the pilosebaceous unit, a population of cells in the upper isthmus has been found to be capable of generating clones in vitro [175,176,177]. Identified by MTS24/PLET-1 antibodies, these cells do not exhibit typical BuSC markers such as K15 or CD34. Another distinct population in the upper isthmus expresses low levels of α6-integrin and is negative for CD34 and SCA-1 markers [178]. These multipotent cells exhibit a unique gene expression pattern compared to BuSCs. The compartment just above the bulge is occupied by a multipotent LGR6^+^ population, contributing to the homeostasis of the IFE and SGs [179]. The overlap between LGR6^+^ with the CD34 and SCA-1 negative population remains unclear.

During HF development, LRIG1^+^ progenitors emerge early in conjunction with presumptive BuSCs and undergo ACDs to form SGs [93,112]. MTS24^+^ progenitors appear to be non-essential for this process [180]. Early embryonic placodes are characterized by LGR6, SHH, and SOX9 markers, with only LGR6^+^ cells giving rise to all the epidermal lineages [179]. Each SC or progenitor population emerges at specific stages during HF development. Nonetheless, the precise connection between embryonic populations and the transition mechanism from embryonic progenitors to adult SCs necessitates further exploration [21]. BLIMP1^+^ cells located in the SG were initially thought to be unipotent SG progenitors regulating SG homeostasis by suppressing c-MYC levels [181]. However, in vivo studies demonstrated that BLIMP1^+^ cells do not exhibit broader cloning capabilities than BLIMP1^−^ cells and lack proliferative properties [182,183]. Instead of representing a sebocyte progenitor pool, BLIMP1 expression identifies terminally differentiated sebocytes. The discovery of these distinct cellular populations poses inquiries about their origins, functions, and potential autonomies, warranting future research and investigation.

The nature of the cells responsible for SG renewal remains unclear. According to the initial hypothesis, SG regeneration is believed not to rely on HFSCs but rather on stem and progenitor cells situated at the periphery of the lobules that are committed to transitioning into the SG lineage [184]. Examinations conducted in mice indicated that paired SGs in HF persist even when one is surgically removed, implying that each lobe is sustained by its SCs [185]. An alternative theory posits that HFSCs play a significant role in SG homeostasis [16,179,186,187]. By genetically marking BuSCs, their distribution along the pilosebaceous unit in mouse skin was monitored, confirming the role of BuSCs in SG homeostasis [188]. The continuous SG regeneration driven by BuSCs was independent of HF regeneration. BuSCs generate progenitors that travel upwards to replenish the SGs and, potentially, the infundibulum. In mouse tail skin, SG renewal by BuSCs occurs over a period of seven days. The initial segment inhabited by BuSC progenitors lies in the basal cell region between the sebaceous duct and the lower tip of the SG. These findings suggest that the lower tip of the SG is primarily responsive to local stimuli that trigger proliferation and differentiation. Upon the activation of a single BuSC, only one of the two sebaceous glands within the same pilosebaceous unit undergoes regular renewal, implying that the simultaneous regeneration of both SGs either necessitates the activation of multiple BuSCs or does not occur concurrently.

Recent research has also corroborated the involvement of HFSCs in developing SGs and the isthmus through comprehensive lineage tracing analyses [189]. The outcomes indicated that the upper BuSCs significantly contribute to the homeostasis of SGs, while the lower BuSCs and HGSCs were not observed to participate in this process. This observation may be attributed to the anatomical proximity of the upper BuSCs to the SGs compared to other HFSC populations. The spatial localization of HFSCs has been shown to influence several of their specialized functions [119,190]. It is noteworthy that recent investigations have identified a population of HFSCs located near the upper HF region adjacent to the SGs [191,192]. This specific population, termed hair follicle-associated pluripotent stem cells (HAPSCs), is characterized as nestin^+^CD34^+^K15^−^ Progenitor cells derived from HAPSCs have been identified within the ORS and demonstrate the ability to differentiate into neural, endothelial, and adipose lineages, indicating their potential to generate sebocytes [189,191,192,193,194,195].

### 4.2. Molecular Drivers Orchestrating SC Behaviour

Molecular cues may have a prominent role in the determination of BuSC fate. Upon reaching the junctional zone, BuSC decides to replenish the SC pool of the infundibulum or contribute to SG homeostasis [188]. The heterogeneous BuSC pool suggests the existence of intrinsic factors influencing BuSC fate, proposing the presence of a BuSC subpopulation with a predilection for generating the SG lineage. Nevertheless, molecular signaling cascades activated by environmental stimuli are believed to be engaged in guiding BuSCs towards the SG lineage. Investigations conducted in *K14ΔNLef1* and *K15ΔNLef1* transgenic mice have illuminated the indispensable role of the β-catenin/Lef1 pathway in establishing LRIG1^+^ and PLET/MTS24^+^ cell populations. Specifically, the expression of the dominant negative form of *Lef1* (*ΔNLef1*) resulted in existing SGs’ hypertrophy and new SGs formed in atypical locations. The ectopic development of SGs was facilitated by the generation of LRIG1^+^ and PLET/MTS24^+^ cells and their respective microenvironments. Intriguingly, the initiation of ectopic SG formation in *K14ΔNLef1* mice was correlated with elevated levels of BuSC markers such as K15, NFATC1, and SOX9. Moreover, SMAD7 has been identified to bind to β-catenin and promote its degradation by recruiting SMURF2, a ubiquitin ligase [196]. The overexpression of *Smad7* in mice has been shown to induce SG generation by inhibiting the Wnt/β-catenin pathway.

The Notch signaling pathway also plays a crucial role in the differentiation towards the SG lineage [185]. *Notch* mutant mice exhibit a loss of SGs, underscoring the essentiality of the Notch cascade involving RBPJ in driving differentiation towards the SG lineage. LRIG1^+^ SCs at the periphery of SGs exhibit heightened RBPJ levels and characteristic Notch signaling activation markers. These markers are particularly abundant in cells situated at the basal region of SGs, marking the site of initial differentiation towards the SG lineage. Furthermore, experiments involving the deletion of the Notch pathway in LRIG1^+^ SCs while sustaining Notch signaling in the bulge and HG revealed that Notch signaling is indispensable for driving the differentiation of LRIG1^+^ cells into SG cells. Through a combination of bioinformatic analyses and functional experiments, it has been demonstrated that the differentiation of sebocytes is associated with the transition from *K14*:*K5* to *K14*:*K79* expression profiles.

**Figure 3:**
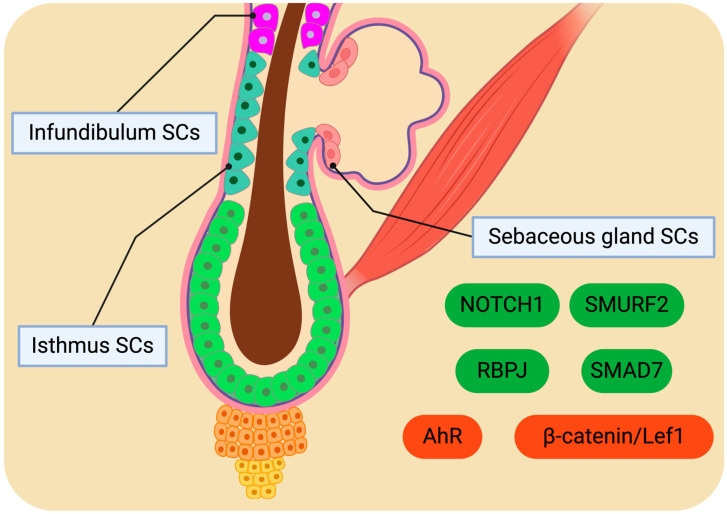
Molecular cues governing the differentiation of sebaceous gland stem cells (SGSCs). Green-colored signals guide SGSC fate towards differentiated sebaceous gland cells, while red-colored signals hinder sebaceous gland generation. The molecular mechanisms responsible for regulating the properties of stem cells located in the infundibulum and isthmus regions have yet to be fully elucidated. Created with BioRender.com.

The AHR is a transcription factor found in progenitor sebocytes that orchestrates the initial phases of xenobiotic metabolism by fostering the expression of cytochrome P450 (CYP1A1) and modulating the levels of BLIMP1 in keratinocytes and sebocytes [197]. Investigations conducted on human skin samples preserved ex vivo and progenitor sebocytes in vitro have unveiled that exposure to TCDD (AHR agonist) results in shortened SGs, hyperplastic sebaceous ducts, restricted expression of sebocyte differentiation markers, and a marked increase in the levels of K10, a keratinocyte differentiation marker [198]. Consequently, TCDD triggers the differentiation of SG progenitors into keratinocyte-like cells rather than sebocytes (Figure 3). It has been postulated that this phenomenon is mediated through the AR signaling cascade. The potential of AHR pathway stimulation to impede sebaceous differentiation by inducing the differentiation of junctional zone progenitors into infundibular cells remains a subject warranting further exploration [199]. All in all, significant inquiries necessitate future research, including the molecular signals provided by the microenvironment that govern the varied responses of BuSCs, as well as the mechanisms underlying the establishment and maintenance of distinct SC populations [188].

## 5. Molecular Profile and Modulation of Sweat Gland SCs

Sweat glands (SwGs) are distributed across the human skin and play a vital role in regulating body temperature [200]. Eccrine SwGs are present throughout the skin, except for the axillae and rectogenital regions where apocrine SwGs are found [201]. Eccrine SwGs comprise a straight duct and a coiled gland structure. The sweat duct (SwD) consists of a basal layer and a suprabasal layer, transitioning into the myoepithelial and luminal layers, respectively, within the coiled gland [202]. Compared to IFE and HFs, SwGs exhibit limited regenerative capacity during homeostasis [203]. Lineage tracing studies in the paw skin of adult mice, the sole region in mouse skin housing SwGs, have demonstrated that the replenishment of SwGs is sustained by a specialized population of SCs termed sweat gland stem cells (SwGSCs); these cells are not present in other epidermal appendages, such as HFs. Unipotent SwGSCs primarily orchestrate the maintenance and regeneration of SwGs following mild injuries, while multipotent SwGSCs assume control of severe trauma cases [203,204].

Unipotent SwSCs comprise four distinct subpopulations known as epidermal, basal, luminal, and myoepithelial SCs, each characterized by unique gene expression profiles and functions [203]. These subpopulations originate from embryonic multipotent SCs. Epidermal SCs, located proximal to the SwD, exhibit high levels of K14 and contribute to wound healing in cases of epidermal injuries. Basal SCs, located along the SwD, express α6-integrin and are responsible for the renewal of the SwD orifice. Luminal SCs, identified by the K19, K18, K15, and CD29 markers, regenerate the luminal layer of the SwG. Lineage tracing studies have revealed that luminal SCs originate from multipotent embryonic epidermal SCs that become unipotent after SwG formation and do not revert to their previous state. Myoepithelial SCs, distinguished by K14 and K5, play a role in SwG homeostasis. Notably, when transplanted into different microenvironments, they exhibit multipotent characteristics, giving rise to SwGs, SwDs, and IFE; however, in vivo, they function solely as unipotent SCs.

Furthermore, LGR6^+^ and BMP1^+^ cells are distributed throughout the acral epithelium [204]. They are recognized as multipotent SCs responsible for the long-term maintenance of SwGs, SwDs, and interadnexal IFE, as well as their regeneration following injury. LGR6^+^ cells demonstrate the rapid proliferation and continuous production of differentiated cells, while BMP1^+^ cells exhibit slow-cycling behavior and sporadic proliferation during homeostasis. After irradiation-induced wounds, BMI1^+^ cells divided rapidly and contributed to the renewal of epithelial tissue, serving as a pool of SCs. LGR5^+^ cells are lineage-restricted progenitors, displaying rapid proliferation and solely regenerating SwGs. A pilot study unveiled the presence of NESTIN^+^ SCs within the stroma of SwGs [205,206]. Upon the isolation and proliferation of human samples in vitro, it was validated that 80% of SwG stroma-derived SCs expressed *NESTIN*. Remarkably, *NESTIN* expression was found to co-occur with Iα6 expression. Comparative analysis with IFESCs and bone marrow SCs revealed a distinctive gene signature pattern in SwG-derived SCs. Furthermore, these cells exhibit cytokine secretion, suggesting their immunological potential to facilitate tissue healing following injuries. These findings underscore the presence of a reservoir of SCs within SwGs possessing multilineage differentiation capacity, rapid proliferation, and self-renewal capabilities. Additionally, NOTCH1 has emerged as a pivotal regulator during embryonic SwG formation, acting as a “gatekeeper” that modulates interactions between SCs and SG lineage-specific niches [207]. Notably, NOTCH1 plays a crucial role in maintaining the stemness of SCs, although it exhibits limitations in dictating cell fates in vitro.

**Figure 4:**
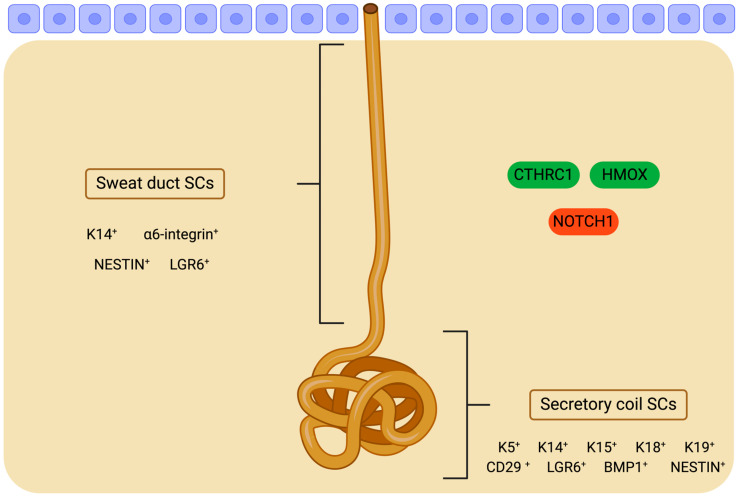
Molecular identity and regulators of sweat gland stem cells (SwGSCs). Green-colored signals guide SCs towards differentiated sweat gland cells, while red-colored signals hinder sweat gland formation. Epidermal SCs, located proximal to the sweat duct, exhibit high levels of K14. Basal SCs, located along the SwD, express α6-integrin. Luminal SCs are characterized by the markers K19, K18, K15, and CD29. Myoepithelial SCs are identified by K14 and K5. Other populations of SwGSCs include those expressing LGR5, LGR6, BMP1, and NESTIN. Created with BioRender.com.

SwGs exhibit constrained regenerative capacity in response to deep wounds, highlighting the need for interventions in patients with deficient SGs [208]. The reconstruction of fully functional skin containing SwGs poses a significant challenge, necessitating the isolation of SwGSCs within the dermis. While previous studies have explored the potential of cellular reprogramming to generate SwG cells from other cell types, these approaches have faced practical limitations in application [208,209,210,211,212,213]. Rigorous investigations have demonstrated the self-renewal and SG regeneration potential of adult human multipotent SwGSCs, offering promising avenues for future research and therapeutic interventions [214,215,216]. However, in vitro, they gave birth exclusively to keratinocytes, indicating that their function depends on the microenvironment [217,218,219]. Three-dimensional (3D) cultures, such as organoids, are reported to substitute SwGSC’s interaction with the microenvironment [220,221,222,223,224,225,226,227,228,229]. CTHRC1 and HMOX were found to induce SwG-specific gene expression synergistically (Figure 4) [230]. The renewing properties of SwGs have been systematically underestimated until recently, although they are considered the major players during epidermis regeneration upon injuries. This is attributed to three factors. Firstly, eccrine SwGs, along with pilosebaceous units, support the reepithelialization of traumas by producing keratinocyte outgrowths that build new IFE. Secondly, keratinocyte outgrowths derived from SwGs are as efficient as those originated by pilosebaceous units. Thirdly, eccrine SwGs are three times more abundant in the human epidermis than pilosebaceous units. Whether eccrine SwGs also participate in IFE renewal during homeostasis requires further investigation [217].

## 6. Conclusions

Skin SCs are located in various regions, including the interfollicular epidermis, bulge, hair germ, infundibulum, isthmus, sebaceous, and sweat glands. A complex network of molecular signals controls the behavior of these skin SCs, maintaining a precise balance between quiescence, self-renewal, and differentiation. The disruption of this intricate equilibrium can lead to SC depletion, impaired wound healing, and the development of conditions such as skin cancer. Future studies may center on unraveling the complexity of signal transduction cascades, epigenetic regulation, and cellular interactions within the skin SC niche, thereby enhancing our comprehension of skin biology and regenerative processes. Moreover, skin SCs offer great promise in regenerative medicine thanks to their remarkable regenerative capabilities and therapeutic potential. Harnessing these cells presents vast opportunities for improved clinical outcomes regarding wound healing, aging, hair follicle regeneration, and cosmetology. Specifically, researchers should endeavor to modulate molecular pathways to augment the regenerative capacity of skin SCs. The development of innovative biomaterials, bioengineered scaffolds, and 3D bioprinting techniques will facilitate the establishment of relevant skin models for drug screening, disease modeling, and personalized medicine applications. Progress in cell-based therapies, encompassing SC transplantation and tissue engineering strategies, holds the potential to revolutionize the field of dermatology by furnishing personalized treatment modalities and regenerative remedies for patients with skin conditions. Thus, an in-depth understanding of the molecular drivers that govern the function of skin SCs paves the way for precise lab-approached manipulations. Collaborative endeavors between laboratory scientists and clinicians will be imperative for translating research discoveries into clinical interventions and propelling state-of-the-art skin SC therapies to the forefront of medical practice. 

## Figures and Tables

**Table 1:** Chromosomal loci of molecular cues implicated in interfollicular epidermal stem cell regulation.

Gene	Chromosomal Locus	Protein	Reference
*ASH1L*	1q22	ASH1-like histone lysine methyltransferase	[82]
*AXIN2*	17q24.1	Axin 2	[64]
*CHRM3*	1q43	Cholinergic receptor muscarinic 3	[95]
*COL17A1*	10q25.1	Collagen type XVII alpha 1 chain	[56,57,58]
*CTNNB1*	3p22.1	Catenin beta 1	[65]
*DLL1*	6q27	Delta-like canonical Notch ligand 1	[63]
*EGFR*	7p11.2	Epidermal growth factor receptor	[77]
*FOS*	14q24.3	Fos proto-oncogene	[59]
*GRHL3*	1p36.11	Grainyhead-like transcription factor 3	[66]
*HDAC1*	1p35.2–p35.1	Histone deacetylase 1	[83]
*IFT88*	13q12.11	Intraflagellar transport 88	[62]
*IRF6*	1q32.2	Interferon regulatory factor 6	[90]
*ITGB1*	10p11.22	Integrin subunit beta 1	[52,54,55,88]
*JUNB*	19p13.13	JunB proto-oncogene	[59]
*KIF3A*	5q31.1	Kinesin family member 3A	[62]
*LAMA3*	18q11.2	Laminin subunit alpha 3	[89]
*LAMB3*	1q32.2	Laminin subunit beta 3	[89]
*LAMC2*	1q25.3	Laminin subunit gamma 2	[89]
*LRIG1*	3p14.1	Leucine-rich repeats and immunoglobulin-like domains 1	[77,92,93]
*MAL*	2q11.1	Mal	[59]
*MYC*	8q24.21	MYC proto-oncogene	[75,77,78,79,80,81]
*NOTCH1*	9q34.3	Notch receptor 1	[63]
*NSUN2*	5p15.31	NOP2/Sun RNA methyltransferase 2	[78]
*PELO*	5q11.2	Pelota mRNA surveillance and ribosome rescue factor	[91]
*RAC1*	7p22.1	Rac family small GTPase 1	[60]
*SFN*	1p36.11	Stratifin	[87]
*SRF*	6p21.1	Serum response factor	[59]
*TAFAZZIN*	Xq28	Tafazzin	[88]
*TGFB1*	19q13.2	Transforming growth factor beta 1	[94]
*TP63*	3q28	Tumor protein p63	[83,84,85,86,87]
*WNT10A*	2q35	Wnt family member 10A	[64]
*WNT4*	1p36.12	Wnt family member 4	[64]
*YAP1*	11q22.1	Yes1 associated transcriptional regulator	[87,88,89]

Data are retrieved from “The Human Protein Atlas” [96].

**Table 2:** Chromosomal loci of molecular drivers implicated in hair follicle stem cell regulation.

Gene	Chromosomal Locus	Protein	Reference
*AXIN2*	17q24.1	Axin 2	[135]
*BMP2*	20p12.3	Bone morphogenetic protein 2	[126]
*BMP5*	6p12.1	Bone morphogenetic protein 5	[126]
*BMP6*	6p24.3	Bone morphogenetic protein 6	[124]
*BMPR1A*	10q23.2	Bone morphogenetic protein receptor type 1A	[158]
*CDH1*	16q22.1	Cadherin 1	[127]
*CDKN1A*	6p21.2	Cyclin-dependent kinase inhibitor 1A	[141]
*CDKN2B*	9p21.3	Cyclin-dependent kinase inhibitor 2B	[141]
*CERS4*	19p13.2	Ceramide synthase 4	[168]
*CTNNB1*	3p22.1	Catenin beta 1	[131,132,144]
*DICER1*	14q32.13	Dicer 1	[163]
*ENG*	9q34.11	Endoglin	[144]
*FGF18*	5q35.1	Fibroblast growth factor 18	[124,125,126]
*FOSB*	19q13.32	FosB proto-oncogene	[167]
*FOXC1*	6p25.3	Forkhead box C1	[126,127]
*FOXI3*	2p11.2	Forkhead box I3	[134]
*FOXP1*	3p13	Forkhead box P1	[125]
*GAΤA6*	18q11.2	GATA binding protein 6	[145]
*HES1*	3q29	Hes family bHLH transcription factor 1	[128]
*JAG1*	20p12.2	Jagged canonical Notch ligand 1	[128,132]
*JUN*	1p32.1	Jun proto-oncogene	[167]
*LEF1*	4q25	Lymphoid enhancer binding factor 1	[132,133]
*LGR4*	11p14.1	Leucine-rich repeat containing G protein-coupled receptor 4	[139]
*LGR5*	12q21.1	Leucine-rich repeat containing G protein-coupled receptor 5	[107,137,138]
*LHX2*	9q33.3	LIM homeobox 2	[150,151]
*LRP6*	12p13.2	LDL receptor-related protein 6	[158]
*MSI2*	17q22	Musashi RNA binding protein 2	[149]
*MTOR*	1p36.22	Mechanistic target of rapamycin kinase	[139]
*MYB*	6q23.3	MYB proto-oncogene, transcription factor	[146]
*MYC*	8q24.21	MYC proto-oncogene	[132]
*NFATC1*	18q23	Nuclear factor of activated T cells 1	[126,148,150,152]
*NOTCH1*	9q34.3	Notch receptor 1	[132]
*OVOL2*	20p11.23	Ovo like zinc finger 2	[147]
*PLA2G2A*	1p36.13	Phospholipase A2 group IIA	[167]
*PRC1*	15q26.1	Protein regulator of cytokinesis 1	[153]
*PRKCI*	3q26.2	Protein kinase C iota	[124]
*RBPJ*	4p15.2	Recombination signal binding protein for immunoglobulin kappa J region	[133]
*RUNX1*	21q22.12	RUNX family transcription factor 1	[140,141,142,143]
*SCD*	10q24.31	Stearoyl-CoA desaturase	[143]
*SFRP1*	8p11.21	Secreted frizzled related protein 1	[135]
*SHH*	7q36.3	Sonic hedgehog signaling molecule	[117,153,154]
*SIRT7*	17q25.3	Sirtuin 7	[148]
*SLC1A3*	5p13.2	Solute carrier family 1 member 3	[48,164]
SMAD1	4q31.21	SMAD family member 1	[133]
SMAD4	18q21.2	SMAD family member 4	[144]
SOAT1	1q25.2	Sterol O-acyltransferase 1	[143]
SOX9	17q24.3	SRY-box transcription factor 9	[150]
TCF3	19p13.3	Transcription factor 3	[131,133]
TCF4	18q21.2	Transcription factor 4	[131,133]
TGFB1	19q13.2	Transforming growth factor beta 1	[144,161]
ZEB1	10p11.22	Zinc finger E-box binding homeobox 1	[147]

Data are retrieved from “The Human Protein Atlas” [96].

## Abbreviations

14-3-3σ	14-3-3 protein sigma
3D	Three-dimensional
ACDs	Asymmetric cell divisions
AhR	Aryl hydrocarbon receptor
aPKC	Atypical protein kinase C
ASH1L	ASH1-like histone lysine methyltransferase
BLIMP1	B lymphocyte-induced maturation protein 1
BMI-1	B cell-specific Moloney murine leukemia virus integration site 1
BMP	Bone morphogenetic protein
BMPR1A	Bone morphogenetic protein receptor type 1A
BuSCs	Bulge stem cells
CD29	Cluster of differentiation 29
CD34	Cluster of differentiation 34
CERS4	Ceramide synthase 4
CHRM3	Cholinergic receptor muscarinic 3
COL17	Type XVII collagen
CTHRC1	Collagen triple helix repeat containing 1
CYP1A1	Cytochrome P450 family 1 subfamily A member 1
DKKs	Dickkopf Wnt signaling pathway inhibitors
DNA	Deoxyribonucleic acid
DP	Dermal papilla
EGFR	Epidermal growth factor receptor
EMT	Epithelial-to-mesenchymal transition
ENG	Endoglin
EPUs	Epidermal proliferative units
EzH2	Enhancer of zeste homolog 2
FGF	Fibroblast growth factor
FOS	Fos proto-oncogene, AP-1 transcription factor subunit
FOXC1	Forkhead box protein C1
FOXI3	Forkead box I3
FOXP1	Forkhead box protein P1
GATA6	GATA binding protein 6
GRHL3	Grainyhead-like transcription factor 3
GTP	Guanosine triphosphate
H2B-GFP	Histone 2B-green fluorescent protein
HAP	Hair follicle-associated pluripotent
HES1	Hairy/enhancer of split-1
HF	Hair follicle
HFSCs	Hair follicle stem cells
HG	Hair germ
HGSCs	Hair germ stem cells
HMOX	Heme oxygenase
IFE	Interfollicular epidermis
IFT88	Intraflagellar transport protein 88 homolog
IRF6	Interferon regulatory factor 6
IRS	Inner root sheath
Iα6	Integrin alpha-5 subunit
JUNB	Transcription factor jun-B
K	Keratin
K15-GFP	K15 promoter-driven green fluorescent protein
KIF3A	Kinesin family member 3A
LEF1	Lymphoid enhancer-binding factor 1
LGR	Leucine-rich repeat containing G protein-coupled receptor
LHX2	LIM homeobox 2
lncRNAs	Long non-coding RNA
LR	Label-retaining
LRIG1	Leucine-rich repeats and immunoglobulin-like domains 1
LRP6	Low-density lipoprotein receptor-related protein 6
MAL	Myelin and lymphocyte protein
miRNA	Micro ribonucleic acid
mRNA	Messenger ribonucleic acid
MSI2	Musashi RNA binding protein 2
mTOR	Mammalian target of rapamycin
NFATC1	Nuclear factor of activated T cells 1
non-LR	Non-label-retaining
NOTCH1	Neurogenic locus notch homolog protein 1
ORS	Outer root sheath
OVOL2	Ovo like zinc finger 2
P450	Cytochrome P450
PAR	Protease-activated receptor
PCR	Polymerase chain reaction
PELO	Pelota mRNA surveillance and ribosome rescue factor
PI3K/Akt	Phosphoinositide-3-kinase–protein kinase B/Akt
PLET1	Placenta expressed transcript 1
PlncRNA-1	Prostate cancer-upregulated long non-coding RNA 1
PORCN	Porcupine O-acyltransferase
PRC1	Protein regulator of cytokinesis 1
RAC1	Ras-related C3 botulinum toxin substrate 1
RBPJ	Recombination signal binding protein for immunoglobulin kappa J region
RNA	Ribonucleic acid
rRNA	Ribosomal ribonucleic acid
RUNX1	Runt-related transcription factor 1
SCA-1	Stem cell antigen 1
SCD1	Stearoyl-CoA desaturase 1
SCDS	Symmetric cell divisions
SCs	Stem cells
SFN	Stratifin
SFRP1	Secreted frizzled-related protein 1
SG	Sebaceous gland
SGSCs	Sebaceous gland stem cells
SHH	Sonic hedgehog
SIRT7	Sirtuin 7
SLC1A3	Solute carrier family 1 member 3
SMAD1	Mothers against decapentaplegic homolog 1
SMAD4	Mothers against decapentaplegic homolog 4
SMAD7	Mothers against decapentaplegic homolog 7
SMURF2	SMAD ubiquitination regulatory factor 2
SOAT1	Sterol O-acyltransferase
SOX9	SRY-box transcription factor 9
sPLA2-IIA	Secreted phospholipase A2 type IIA
SRF	Serum response factor
SwD	Sweat duct
SwGs	Sweat glands
SwGSCs	Sweat gland stem cells
TACs	Transit-amplifying cells
TARBP2	TAR (HIV) RNA-binding protein 2
TAZ	Tafazzin
TCDD	2,3,7,8-Tetrachlorodibenzo-p-dioxin
TCF3	Transcription factor 3
TCF4	Transcription factor 4
Tet-o-H2B-GFP	“Tet On” regulated expression of the H2B-GFP fusion protein
TGFβ	Transforming growth factor β
YAP	Yes-associated protein
ΔΝp63α	Delta Np63 alpha
